# *NOTCH1* mutation associates with impaired immune response and decreased relapse-free survival in patients with resected T1-2N0 laryngeal cancer

**DOI:** 10.3389/fimmu.2022.920253

**Published:** 2022-07-15

**Authors:** Xiao-yang Gong, Hai-bin Chen, Li-qing Zhang, Dong-sheng Chen, Wang Li, Dong-hui Chen, Jin Xu, Han Zhou, Le-le Zhao, Yun-jie Song, Ming-zhe Xiao, Wang-long Deng, Chuang Qi, Xue-rong Wang, Xi Chen

**Affiliations:** ^1^ Department of Otorhinolaryngology, The First Affiliated Hospital, Nanjing Medical University, Nanjing, China; ^2^ Jiangsu Simcere Diagnostics Co., Ltd, The State Key Laboratory of Translational Medicine and Innovative Drug Development, Nanjing, China; ^3^ Department of Pharmacology, Nanjing Medical University, Nanjing, China

**Keywords:** T1-2N0 laryngeal cancer, relapse, tumor mutation burden, *NOTCH1* mutation, tumor immunity, molecular alterations

## Abstract

**Background:**

Patients with early-stage laryngeal cancer, even stage T1-2N0, are at considerable risk of recurrence and death. The genetic and immunologic characteristics of recurrent laryngeal cancer remain unclear.

**Methods:**

A total of 52 T1-2N0 laryngeal cancer patients were enrolled. Of these, 42 tissue samples were performed by targeted DNA sequencing, and 21 cases were performed by NanoString immuno-oncology targeted RNA sequencing to identify the distinct molecular bases and immunologic features associated with relapse in patients with early laryngeal cancer, respectively.

**Results:**

To the best to our knowledge, we present for the first time an overview of the genomic mutation spectrum of early-stage laryngeal cancers. A total of 469 genomic alterations were detected in 211 distinct cancer-relevant genes, and the genes found to be mutated in more than five patients (>10%) included *tumor protein p53* (*TP53*, 78.5%), FAT atypical cadherin 1 (*FAT1*, 26%), LDL receptor related protein 1B (*LRP1B*, 19%), cyclin dependent kinase inhibitor 2A (*CDKN2A*, 17%), tet methylcytosine dioxygenase 2 (*TET2*, 17%), notch receptor 1 (*NOTCH1*, 12%) and neuregulin 1 (*NRG1*, 12%). Recurrent laryngeal cancer demonstrated a higher tumor mutation burden (TMB), as well as higher *LRP1B* mutation and *NOTCH1* mutation rates. Univariate and multivariate analyses revealed that high TMB (TMB-H) and *NOTCH1* mutation are independent genetic factors that are significantly associated with shorter relapse-free survival (RFS). Simultaneously, the results of the transcriptome analysis presented recurrent tumors with *NOTCH1* mutation displayed upregulation of the cell cycle pathway, along with decreased B cells score, T cells score, immune signature score and tumor-infiltrating lymphocytes (TILs) score. The Cancer Genome Atlas (TCGA)-laryngeal cancer dataset also revealed weakened immune response and impaired adhesion functions in *NOTCH1*-mutant patients.

**Conclusions:**

Genomic instability and impaired immune response are key features of the immunosurveillance escape and recurrence of early laryngeal cancer after surgery. These findings revealed immunophenotypic attenuation in recurrent tumors and provided valuable information for improving the management of these high-risk patients. Due to the small number of patients in this study, these differences need to be further validated in a larger cohort.

## Introduction

Laryngeal cancer, which mostly arises in either the supraglottic or glottic regions, accounts for one-third of all head and neck cancers and may be a significant contributor to morbidity and mortality (1). For early-stage laryngeal cancers, including T1-2N0 lesions, surgical treatment is preferred due to its high cure rate of nearly 90% (2). However, the postoperative local recurrence rate of stage T1 glottic cancers is 5-10%, and that of early supraglottic cancers is 10-20% (1). Therefore, it is of great significance to clarify the genomic and immunological characteristics of relapse mechanisms so that more accurate treatment strategies can be offered to patients with early laryngeal cancer at high risk.

Genetic alterations in cancer cells are associated with tumor progression and metastasis, and there is evidence that *KRAS*, epidermal growth factor receptor (*EGFR*) and *TP53* mutations are associated with relapse or worse survival outcomes in patients with early lung adenocarcinoma (3, 4). Aberrant NOTCH signaling has been associated with cancers multiple biological processes, such as carcinogenesis, invasion, and angiogenesis (5, 6). Specifically, *NOTCH1* mutation has been proven to be associated with invasion and metastasis in advanced head and neck squamous cell carcinomas (HNSCC) (7). Beyond individual somatic gene mutations, TMB has attracted attention as an indicator of immune checkpoint inhibitor efficacy (8, 9). Moreover, TMB has also been verified to be significantly higher in the recurrent group in resected non-small-cell lung cancer (NSCLC) and in locally advanced triple-negative breast cancer (10, 11). On account of the understanding of the mutational landscape of early-stage laryngeal cancers is limited, the research progress on laryngeal cancer recurrence and the association with genomic mutations has been minimal, and more studies are urgently needed.

In addition to genetically defined biomarkers, the tumor immune microenvironment (TiME) plays a key role in tumorigenesis and cancer progression by closely interacting with the tumor cells, and the immune system is crucial to the cancer onset and evolution (12–14). The TiME is composed of cellular and non-cellular components, while the cancer cells, fibroblasts and immune cells in this niche are mainly composed of cellular components. Cytokines, chemokines and growth factors mainly constitute the non-cellular components (15). TILs have been proposed as crucial prognostic indicators in several cancer types (16–18), and the TIL density has greater predictive power for survival than the well-established tumor, node, metastasis (TNM) classification system in gastric cancer (19). In a study exploring the TiME in laryngeal cancer, Alessandrini et al. determined that the presence of tertiary lymphoid structures is a positive prognostic factor and that programmed cell death ligand 1 (PD-L1) appears to be an indirect marker of an effective anti-tumor response in laryngeal cancers (20). Notably, advances in cancer research have highlighted the importance of the immune system in controlling tumor progression and metastasis (21). However, the relationship between the TiME and the postoperative recurrence of early laryngeal cancer is still poorly studied.

To this end, we performed targeted DNA-based sequencing to comprehensively investigate differences in the genomic profiles between relapsed and non-relapsed tumors from patients with T1-2N0 laryngeal cancer. Further, an immune oncology panel-related RNA sequencing was used to investigate the TiME between relapsed tumors with and without *NOTCH1* mutation. By analyzing the sequencing data, the molecular landscape of relapsed tumors was characterized in detail, and the immunologic features of the TiME were elucidated, which may contribute to new insights into the mechanisms by which cancer cells evade immune surveillance and improve the individual management of patients experiencing laryngeal cancer relapse.

## Methods

### Patient enrollment and ethical compliance

In total, 52 patients who were diagnosed with stage T1-2N0 primary laryngeal cancer were retrospectively and continuously collected from January, 2017 to January, 2020. All patients received laryngectomy without neo- or adjuvant chemotherapy or other systemic therapies, and all patients were followed up from the time of diagnosis until January 2022. A diagnosis of recurrence required clinical confirmation by computed tomography and magnetic resonance imaging exams. The collected formalin-fixed, paraffin-embedded (FFPE) samples were sent to Nanjing Simcere Diagnostics Co., Ltd. (Nanjing, China) for targeted DNA-based next-generation sequencing (NGS) analysis by TruSight Oncology 500 (TSO500) and NanoString RNA gene expression assays.

**Figure 1:**
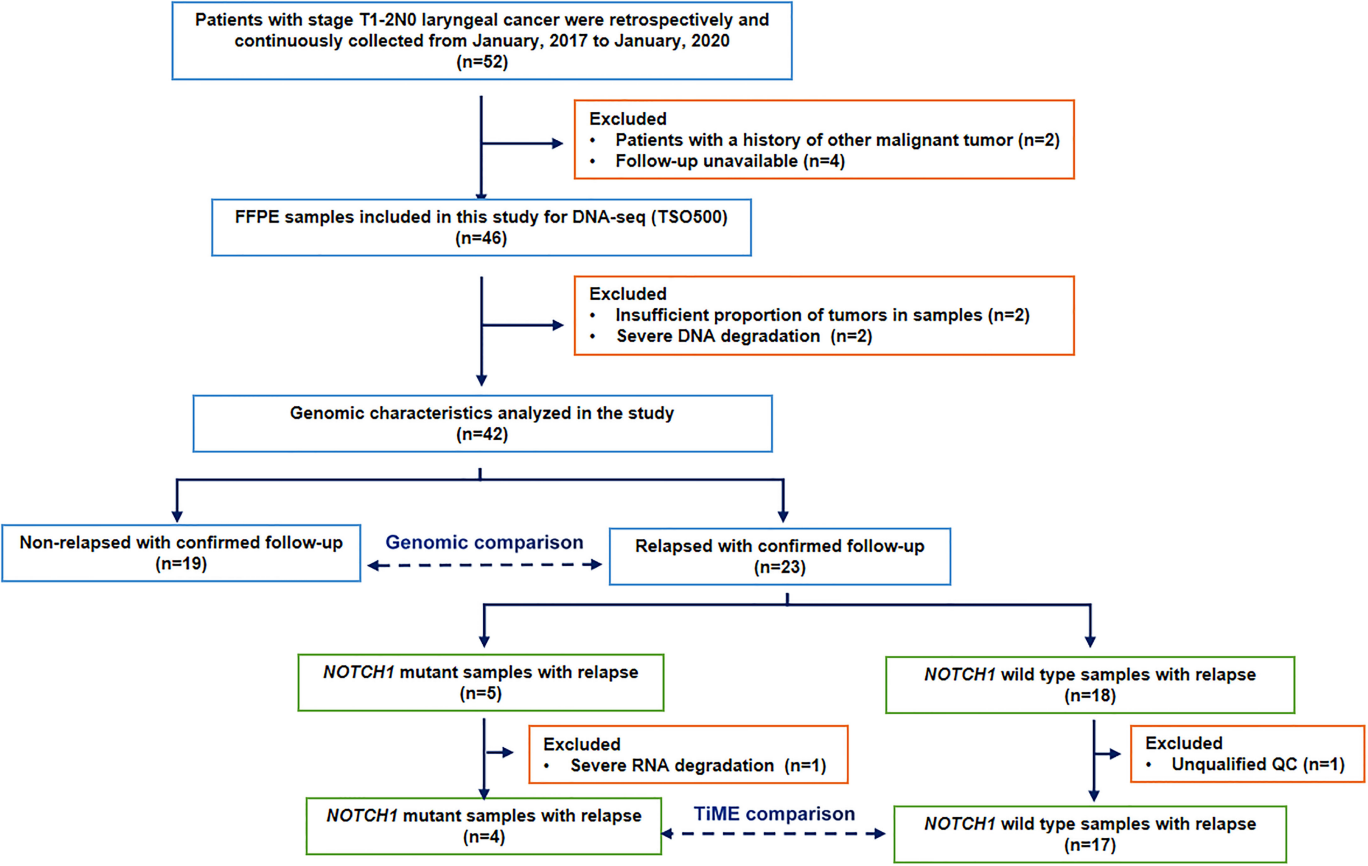
Flowchart of patient selection.

Considering the median time between primary diagnosis and local recurrence was 1.7 years (range 0.6–8.9 years) in 38 relapsed patients of total 303 patients with T1 glottic cancer (22), and the postoperative local recurrence rate of stage T1 supraglottic cancers is slightly higher than glottic cancers (1). Together with our clinical observations, we chosed 24 months as the relapse threshold in resected T1-2N0 laryngeal cancer to discuss the causes and mechanisms of rapid postoperative recurrence in our study. Thus, during the postoperative follow-up period, patients with local or distant recurrence within 24 months were classified into the relapsed group (n=23), and those without local recurrence more than 2 years after surgery were classified into the non-relapsed group (n=19). After excluding unqualified and unavaliable follow-up specimens, 42 postoperative FFPE samples were included in the molecular characterization analysis. 21 relapsed samples with qualified quality control were analyzed for immunologic features according to specifically defined criteria. The patient selection flowchart is shown in **Figure 1**. This study was approved by the (blinded for peer review).

### DNA extraction and library preparation

For clinical FFPE samples used for TSO500 evaluation, a minimum of 20% tumor content is needed. Genomic DNA (gDNA) was extracted from tissues using a tissue sample DNA extraction kit (Kai Shuo). All extraction procedures were carried out according to the manufacturer’s protocols. The DNA samples were captured and enriched by a two-step specific capture probe, and the DNA library and corresponding cDNA library were standardized by the library homogenization method, purified by magnetic beads, and sequenced using the Illumina NextSeq 550Dx platform. The sequencing results were analyzed by the TSO500 Docker pipeline. TSO500 NGS libraries were prepared using the TSO500 DNA Kit. Library preparation was performed manually according to the manufacturer’s protocol, with 24 samples per batch.

### Library sequencing and bioinformatics analysis

Prior to library normalization, the NGS libraries enriched by hybridization capture were quantified using the Qubit dsDNA HS Assay Kit. NGS sequencing was performed on the NextSeq 550 instrument (Illumina) with 8 libraries per sequencing. Base calls obtained through Illumina NovaSeq were converted to FASTQ files. The fastp tool (v.2.20.0) was used for adapter pruning and to filter low-quality substrates (23). The internal database was used to screen for germline variations. Copy number variation (CNV) involves amplification and deletion identified by CRAFT copy number callers from the TSO500 pipeline. Manta (Version 1.6.0) is used to detect large-scale structural variation (SV) in RNA libraries, and only fusions with at least three unique reads, one of which being a split read crossing a fusion break point, are considered candidate fusions (24). After screening for germline variations (internal database) and using the high COSMIC database to count mutations, the number of eligible somatic mutations (coding or high confidence regions, more than 50 times coverage, more than 5% VAF single nucleotide variations (SNVs) and INDELs were measured per megabase (Mb) by NGS (25). TMB measurements consider only SNV and INDELs in the coding region. Raw sequencing data and the TMB were evaluated using Illumina TSO500 support software. Based on the top 25% of the TMB scores, the 42 patients were divided into TMB-H and TMB-Low (TMB-L) groups. The TSO500 Docker pipeline was used to determine the sample microsatellite status in the form of a microsatellite instability (MSI) score. Samples with MSI scores ≥ 20 were considered microsatellite instable, and the rest were considered microsatellite stable.

### Transcriptional profiling

Total RNA was isolated from 5-μm FFPE slices using the Qiagen RNeasy FFPE Kit, and 100 ng of RNA was hybridized to a version of the NanoString PanCancer code set for reading on the nCounter platform (NanoString). The expression of 289 immune-related genes, including housekeeping genes, was assessed (**sTable 1**). Using nSolver 2.6 software, housekeeping genes were used to normalize the expression values as recommended by the manufacturer. According to the manufacturer’s specification, the genes are divided into 14 immune cell types (T cells, B cells, mast cells, dendritic cells, macrophages, neutrophils, cytotoxic cells, exhausted CD8 cells, NK-CD56 cells, CD8 T cells, CD45 cells Th1 cells, NK cells and Treg cells) (**sTable 2**). Meagene scores were calculated based on the geometric mean expression levels of member genes (26). We also studied 10 previously published prognostic and immunotherapy response meta-genes. We calculated these metagene scores using the methods described in the respective publications (**sTable 3**).

### DEGs and functional enrichment analysis

Differentially expressed genes (DEGs) were selected based on the *NOTCH1* mutation status of relapsed laryngeal cancer patients through the “DEseq2” software package, with log2 |fold change| >1 and False Discovery Rate (FDR) <0.05. Heatmaps of DEGs were made using the “HeatMap” package. We then used Gene Ontology (GO) and Kyoto Encyclopedia of Genes and Genomes (KEGG) analysis to analyze the possible biological processes in which the overlapping DEGs are involved. Also, genomic enrichment analysis (GSEA) analysis was used to further explore gene expression differences in laryngeal cancer patients.

### Statistics

Continuous data with a normal distribution were presented as means and standard deviations and that with a screwed distribution were presented as medians and ranges. Categorical data were presented as frequencies or percentages. Differences between independent groups were statistically measured using the Wilcoxon Mann-Whitney test. Survival curves were drawn using the Kaplan-Meier method. Relapse-free survival (RFS) time was defined as the time between the diagnosis and confirmed disease relapse of the patient. Overall survival (OS) time was defined as the time between the diagnosis and death of the patient. A *p*-values < 0.05 was considered statistically significant. All statistical analyses were performed using the Graphpad (V 10.0), R (V. 4.1.0) and R Bioconductor packages (https://www.r-project.org).

## Results

### Patient characteristics

Data from 52 patients with stage T1-2N0 laryngeal cancer were retrospectively and continuously collected from January, 2017 to January, 2020. Two patients with a history of other malignant tumor and four patients with unavailable follow-up were excluded. Besides, in the process of DNA-sequencing, two samples without sufficient proportion of tumors and another two samples with severe DNA degradation were also excluded. Finally, there were 42 patients (37 males and 5 females) analyzed in the study, with a median age of 67 years (40 to 87 years). The proportion of patients with stage T1 was 83% (35/42). Other than the TMB value, there were no significant differences between the two groups terms of in sex, age, smoking history, drinking history, clinical stage or anterior involvement. The clinicopathological features of the patients are shown in **Table 1**.

**Table 1:** The clinicopathological features of the patients.

Characteristic	Non-relapsed, n=19	Relapsed, n=23	p-value^1^
**Sex**			>0.9
Female	2 (11%)	3 (13%)	
Male	17 (89%)	20 (87%)	
**Age**			0.3
Mean (SD)	65 (9)	68 (12)	
**Somking_history**			0.5
Non-smoker	10 (53%)	10 (43%)	
Light smoker	4 (21%)	9 (39%)	
Heavy smoker	5 (26%)	4 (17%)	
**Drinking_history**			0.8
Never	10 (53%)	13 (57%)	
Former	4 (21%)	3 (13%)	
Always	5 (26%)	7 (30%)	
**Stage**			0.11
T1	18 (95%)	17 (74%)	
T2	1 (5.3%)	6 (26%)	
**Anterior commissure involvement**	9 (47%)	15 (65%)	0.2
**TMB** Median (IQR)	4.69 (3.12, 5.86)	7.03 (4.30, 7.81)	0.045

### Overview of the genomic mutation spectrum

**Figure 2:**
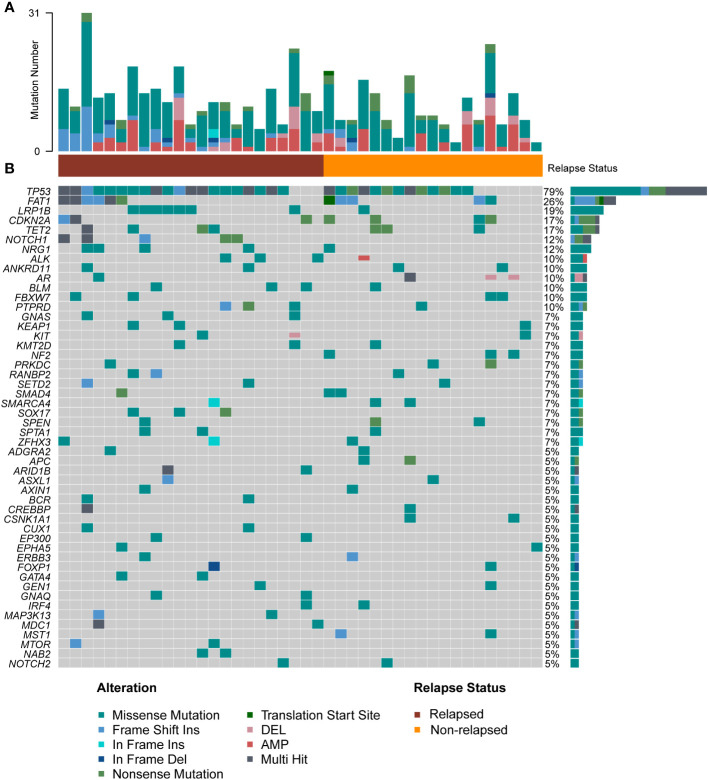
Overall level mutation analysis of laryngeal cancer patients. **(A)** No. of genomic alterations in each patient. **(B)** A waterfall map of the genetic mutations in the study, light gray color in the genetic mutation waterfall diagram correspond to the wild type or absence of any mutation.

We performed targeted NGS of 523 cancer-relevant genes (**sTable 4**) using treatment-naïve tumor biopsies from 42 laryngeal cancer patients. **Figure 2A** shows that the number of mutations per patient ranged from 2 to 31, with a median of 7.0. The mean number of mutations in the relapsed group was higher than that in the non-relapsed group (9.0 vs. 6.0, p=0.045), indicating that more somatic mutations were present in the former group. **Figure 2B** depicts the genetic changes in the entire cohort, a total of 469 genomic alterations were detected in 211 distinct cancer-relevant genes, and 51 mutated genes (>5% patients) within the 523-gene panel are shown. The genes found to be mutated in more than five patients (>10%) included *TP53* (78.5%), *FAT1* (26%), *LRP1B* (19%), *CDKN2A* (17%), *TET2* (17%), *NOTCH1* (12%) and *NRG1* (12%). All samples presented microsatellite stable status.

**Figure 3:**
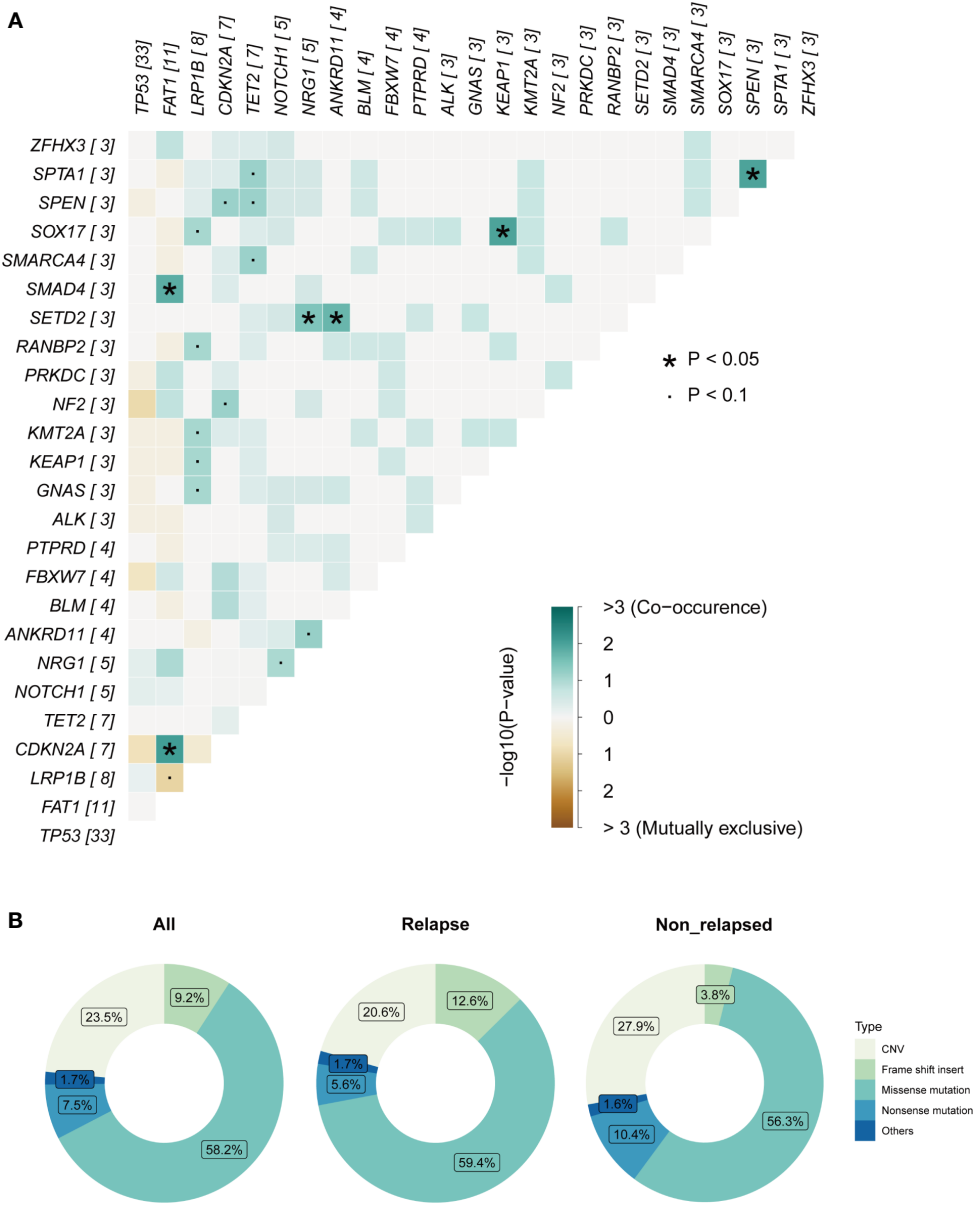
Mutation analysis of laryngeal cancer patients. **(A)** Pairwise mutual exclusivity and co-occurrence analysis in the cohort. **(B)** Separate group analyses showed no significant difference in the proportion of mutation types between the two defined group.

Among these genes, *TP53*, a gene related to DNA repair, displayed the highest mutation frequency in the cohort, and the most common mutation type was mis-sense mutation (**Supplementary Figure 1**). Patients in the relapsed group tended to have a higher *TP53* mutation rate (87% vs. 68%). The second most commonly mutated gene was *FAT1*, a tumor suppressor preventing cancer development, *FAT1* promotes the migration of HCC cells by regulating the expression levels of EMT-associated genes (27). The major mutation type in our cohort was frame-shift mutation (**Supplementary Figure 1**). Interestingly, pairwise mutual exclusivity and cooccurrence analysis revealed that *FAT1/CDKN2A*, and *FAT1/SMAD family member 4 (SMAD4)* were significantly co-occurring (p<0.05), and *FAT1* typically displayed exclusivity with *LRB1P* (p<0.1) (**Figure 3A**). *LRP1B*, which encoding endocytic LDL-family receptor, is among the top 10 significantly mutated genes in human cancer (28). *LRP1B* mutation is associated with a higher TMB and better survival outcomes in patients with melanoma and NSCLC (29). In our study, the only type of mutation found in *LRP1B* was missense mutation (**Supplementary Figure 1**), and *LRB1P* mutation tended to co-occur with GNAS complex locus *(GNAS)*, kelch like ECH associated protein 1 *(KEAP1)*, lysine methyltransferase 2D *(KMT2D)*, RAN binding protein 2 *(RANBP2)* and SRY-box transcription factor 17 *(SOX17)* mutation (p<0.1) (**Figure 3A**). In addition, cell cycle related gene *CDKN2A*, tumor suppressor genes like *TET2*, *NOTCH1* and *NRG1* were all found to have a high mutation percentage in the cohort. Of note, all patients with *NOTCH1* mutations were in the relapsed group (**sFigure 1**). Co-occurring mutations in *SETD2/NRG1*, *SETD2/* ankyrin repeat domain containing 11 (*ANKRD11)*, *SOX17/* kelch like ECH associated protein 1 (*KEAP1*) and spectrin alpha, erythrocytic 1 (*SPTA1*)*/spen family transcriptional repressor* (*SPEN*) were also found (p<0.05) (**Figure 3A**). Overall, a total of five major mutation types were detected: 273 mis-sense mutations (58.2%), 110 copy number variations (23.4%), 43 frame-shift mutations (9.2%), 35 nonsense mutations (7.5%) and 8 other mutation types (1.7%). Separate group analyses showed no significant difference in the proportion of mutation types between the two defined groups (**Figure 3B**).

### TMB-H and *NOTCH1* mutation were related to decreased RFS

**Figure 4:**
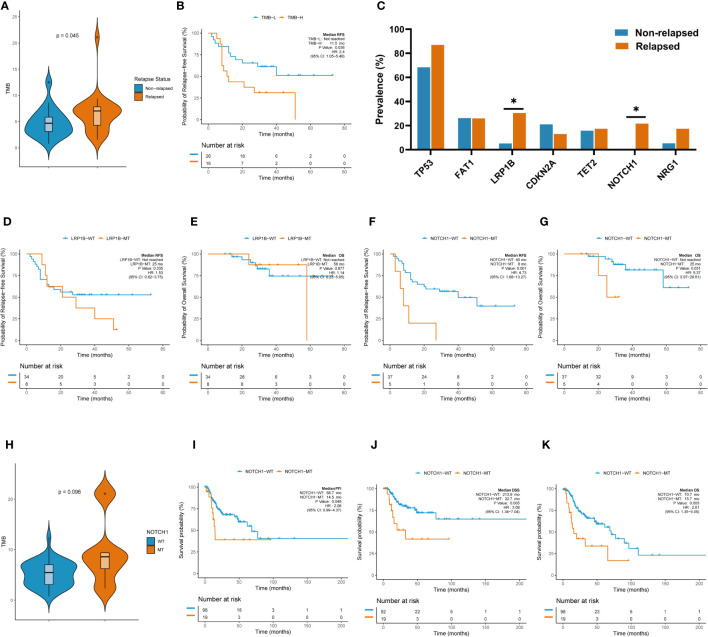
TMB and individual gene mutations were associated with clinicalsurvival outcomes. **(A)** TMB values in the relapsed group and the non-relapsed group. **(B)** Higher TMB (top 25%) was associated with poorer RFS outcomes. **(C)** Rate of individual somatic mutated genes between the two groups. **(D)** K-M survival curve on *LRB1P* mutation with RFS. **(E)** K-M survival curve on *LRB1P* mutation with OS. **(F)** K-M survival curve on *NOTCH1*mutation with RFS. **(G)** K-M survival curve on *NOTCH1* mutation with OS.**(H)**TMB values in the *NOTCH1* mutation group and wild-type group. **(I)** K-M survival curve on *NOTCH1* mutation with PFI in TCGA. **(J)** K-M survival curve on *NOTCH1* mutation with DSS in TCGA. **(K)** K-M survival curve on *NOTCH1* mutation with OS in TCGA.

TMB is thought to be related to the number of neoantigens in tumors and plays an important role in predicting the efficacy of immunotherapy (8, 9). TMB-H has been shown to be a poor prognostic factor for recurrence in patients with resected NSCLC. Therefore, we investigated whether TMB-H is associated with decreased RFS in the laryngeal cancer cohort. Interestingly, the TMB values in the relapsed group were higher than those in the non-relapsed group (7.03 vs. 4.69, p=0.045, **Figure 4A**), suggesting that there were more somatic gene mutations in the relapsed group. In line with the findings reported for resected NSCLC, a higher TMB (top 25%) was associated with poorer RFS outcomes in the resected laryngeal cancer cohort (median RFS: not reached vs. 11.5 months in TMB-L vs. TMB-H; Hazard ratio (HR): 2.4, 95% CI: 1.05-5.48, p=0.036; **Figure 4B**). Next, we further examined the clinical impact of the presence of multiple somatic mutations (>1 mutation) and only one somatic mutation in this cohort. In line with the findings for TMB-H, patients with multiple somatic gene mutations (>1 mutation) (n=37) showed worse RFS than those (n=5) with only one somatic gene mutation (p=0.033) (**sFigure 1B**). These results indicate that TMB-H, which is characterized by a high number of somatic gene mutations, may be related to the degree of malignancy of laryngeal cancer, leading early recurrence in laryngeal cancer.

We further explored the rate of individual somatic mutated genes between the two groups. Among all the candidate genes (mutated in more than five cases, >10% patients), *LRB1P* mutation (30.4% vs. 5.3%, p=0.039, **Figure 4C**) and *NOTCH1* mutation (21.7% vs. 0%, p=0.030, **Figure 4C**) were occurred more frequently in the relapsed group, with significant differences observed. Specifically, all *NOTCH1* mutations occurred in the relapsed group. However, Kaplan–Meier survival curves showed that *LRB1P* mutation was not associated with RFS (median RFS: not reached vs. 25.0 months in *LRB1P*-WT vs. *LRB1P*-Mu; HR: 1.53, 95% CI: 0.62-3.75, p=0.335; **Figure 4D**) or overall survival (OS) (median OS: not reached vs. 58.0 months in *LRB1P*-WT vs. *LRB1P*-Mu; HR: 1.14, 95% CI: 0.23-5.65, p=0.877; **Figure 4E**) in our laryngeal cancer cohort. Of note, patients with *NOTCH1* mutation presented significantly shorter RFS (median RFS: 40.0 vs. 8.0 months in *NOTCH1*-WT vs. *NOTCH1*-Mu; HR: 4.73, 95% CI: 1.68-13.27, p=0.001; **Figure 4F**) and OS (median OS: not reached vs. 25 months in *NOTCH1*-WT vs. *NOTCH1*-Mu; HR: 5.37, 95% CI: 0.97-29.61, p=0.031; **Figure 4G**). In addition, TMB values in the *NOTCH1*-mutant group were higher than those in the wild-type group, although this difference was not significant, possibly due to the small number of samples (9.5 vs. 5.2, Mu vs. WT, p=0.098). Consistent with the findings in our cohort, the TCGA laryngeal cancer dataset confirmed that patients with *NOTCH1* mutation across different tumor stages, mainly advanced stages, had significantly shorter progression-free interval (PFI) (median PFI: 68.7 months vs. 14.5 months in *NOTCH1*-WT vs. *NOTCH1*-Mu; HR: 2.08, 95% CI: 0.99-4.37, p=0.049; **Figure 4I**), disease-specific survival (DSS) (median DSS: 213.9 months vs. 32.7 months in *NOTCH1*-WT vs. *NOTCH1*-Mu; HR: 3.08, 95% CI: 1.34-7.04, p=0.005; **Figure 4J**) and OS (median OS: 70.7 months vs. 15.7 months in *NOTCH1*-WT vs. *NOTCH1*-Mu; HR: 2.61, 95% CI: 1.35-5.05, p=0.003; **Figure 4K**). These results suggest that *NOTCH1* mutation is a poor prognostic factor for laryngeal cancer and that patients with resected laryngeal cancer whose tumors carried *NOTCH1* mutation have a higher risk of relapse.

### The independent prognostic role of *NOTCH1* mutation in disease recurrence and survival

Clinical factors and genetic alterations are potential predictors of prognosis; therefore, we used univariate and multivariate Cox regression models to examine the correlation between genetic changes and patients’ RFS and OS. Baseline clinical characteristics including sex, age, smoking history, drinking history, clinical stage and anterior commissure involvement status were also examined. The results of univariate analysis showed that *NOTCH1* mutation (HR: 4.44, 95% CI: 1.59-12.4, p=0.004) and TMB-H (HR: 2.40, 95% CI: 1.05-5.48, p=0.038) were adverse prognostic factors for RFS (**Table 1**). To determine whether *NOTCH1* mutation and TMB-H were independent predictors of survival outcomes, variables with p<0.05 in the univariate analysis were then included in multivariate analysis. The results of multivariate analysis showed that both *NOTCH1* mutation (HR: 4.18, 95% CI: 1.20-14.6, p=0.025) and TMB-H (HR: 2.85, 95% CI: 1.16-7.01, p=0.023) were independent genetic factors found to be significantly associated with shorter RFS (**Tables 1**, **2**). In addition, univariate and multivariate analyses revealed that only *NOTCH1* mutation was identified as an independent prognostic factor for OS (univariate, HR: 5.37, 95% CI: 0.97-23.6, p=0.054; multivariate, HR: 10.2, 95% CI: 1.49-69.7, p=0.018) (**sTable 5**).

**Table 2:** The results of univariate and multivariate analysis on NOTCH1 mutation with RFS.

Univariable				Multivariable			
Characteristic	HR^1^	95% CI^1^	p-value	HR^1^		95% CI^1^	p-value
Sex							
Female	—	—		—		—	
Male	0.94	0.28, 3.20	>0.9				
Age_group							
< 65	—	—		—		—	
>= 65	1.82	0.74, 4.43	0.2				
Somking_history							
Non_smoker	—	—		—		—	
Light_smoker	2.00	0.81, 4.96	0.13	1.59		0.59, 4.32	0.4
Heavy_smoker	0.85	0.27, 2.71	0.8	0.48		0.12, 1.90	0.3
Drinking_history							
Never	—	—		—		—	
Former	0.60	0.17, 2.11	0.4				
Always	1.02	0.41, 2.56	>0.9				
Stage							
T1	—	—		—		—	
T2	2.21	0.87, 5.65	0.10	1.94		0.67, 5.65	0.2
Anterior_commissure_involvement							
No	—	—		—		—	
Yes	1.48	0.63, 3.50	0.4				
LRP1B							
WT	—	—		—		—	
MU	1.53	0.62, 3.75	0.4				
NOTCH1							
WT	—	—		—		—	
MU	4.44	1.59, 12.4	0.004	4.18		1.20, 14.6	0.025
TMB_level							
TMB-L	—	—		—		—	
TMB-H	2.40	1.05, 5.48	0.038	2.85		1.16, 7.01	0.023

^1^HR = Hazard Ratio, CI = Confidence Interval.

### Identification of DEGs based on *NOTCH1* mutation

**Figure 5:**
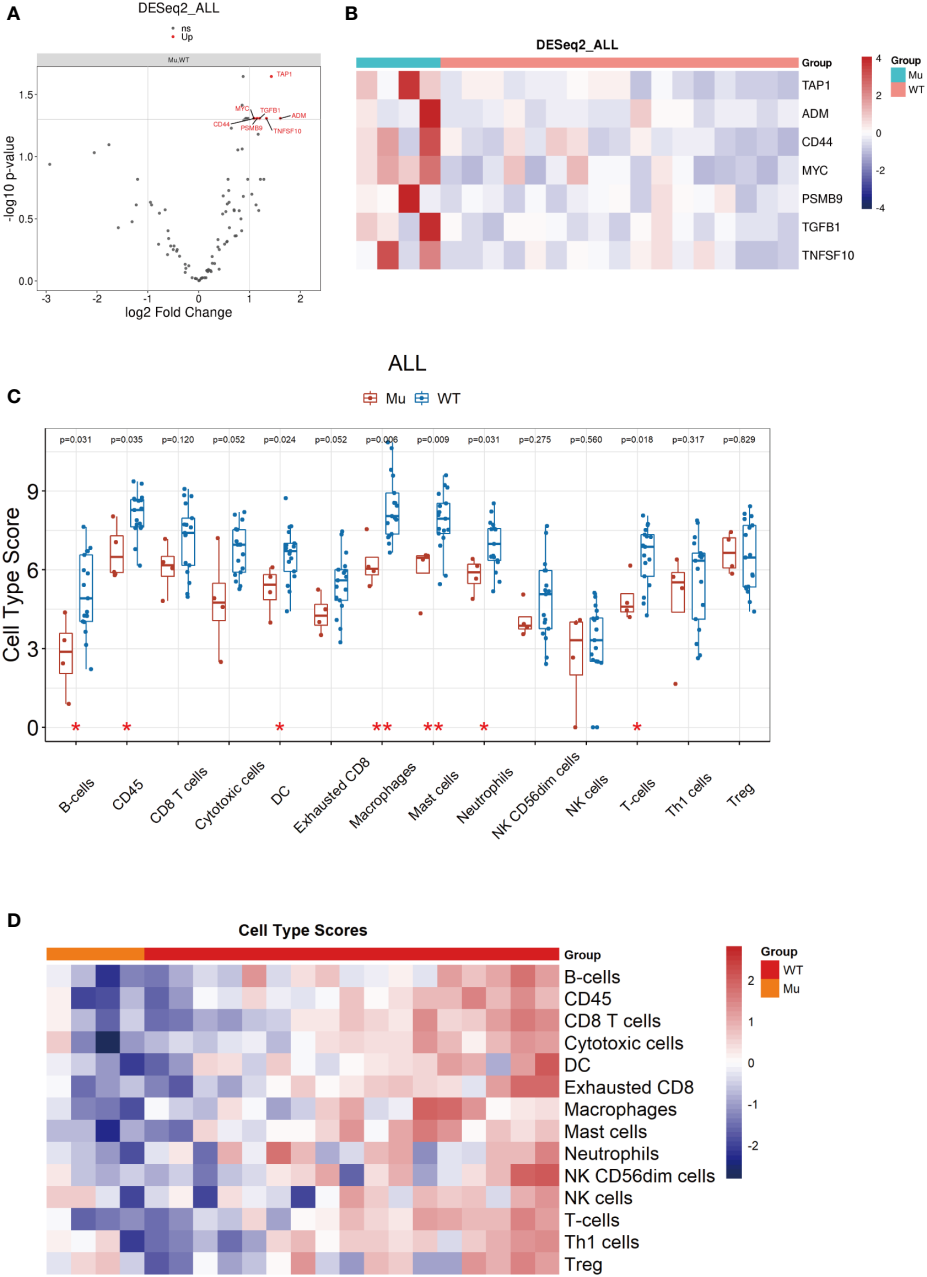
Identification of DEGs based on *NOTCH1* mutation and immune cell profile analyses. **(A, B)** DEGs were identified and shown by volcano plot and heatmap. **(C, D)** The immune cell composition assessment shown as heatmap and the scores of discrepant immune cell subsets in two groups.

Previous studies suggested that a weakened immune phenotype was present in relapsed stage IA NSCLC tumors (30, 31). Thus, to elucidate the role of *NOTCH1* mutation on recurrence in resected laryngeal cancers, we further investigated the variation in the anti-tumor immunity of the TiME in relapsed tumors with *NOTCH1* mutation and *NOTCH1* wild-type tumors by Nano-String gene expression assay. After excluding specimens exhibiting RNA degradation or unqualified quality control, tumors from 4 *NOTCH1* mutation patients with relapse and 17 *NOTCH1* wild-type relapsed patients were included in the analysis. We first explored the DEGs between the two groups. In total, seven upregulated DEGs were identified in *NOTCH1* mutation group, which are shown as a heatmap and volcano plot (**Figures 5A, B**). In DEGs, most of these genes were related to specific growth-stimulating molecules, such as *MYC* (MYC proto-oncogene), *ADM* (adrenomedullin), *TGFβ1* (transforming growth factor beta 1) and *TAP1* (transporter associated with antigen processing 1). Besides, the expression of *CD44*, a cell-surface glycoprotein gene involved in cell-cell interactions, cell adhesion and migration, was higher in patients with *NOTCH1* mutation. The expression of *TNFSF10* (TNF superfamily member 10) and *PSMB9* (proteasome 20S subunit beta 9) were also higher in patients with *NOTCH1* mutation. In particular, these upregulated genes were determined to have the potential to be small-molecule targets for precision therapy in patients who had recurrent laryngeal cancer with *NOTCH1* mutations.

### Pathway and immune cell profile analyses

Furthermore, we explored the function of DEGs by GO and KEGG analysis and found that the enrichment of DEGs by GO was mainly concentrated in receptor ligand activity, signaling receptor activator activity pathways in molecular function (**Supplementary Figure 2A**). KEGG analysis showed that DEGs enrichment was concentrated in Natural killer cell-mediated cytotoxicity and cell adhension molecules pathways (**Supplementary Figure 2B**). In addition, the GSEA results demonstrated that cell cycle pathway were enriched in the *NOTCH1* mutation group (NES: 1.865, p.adjust=0.004), indicating that *NOTCH1* mutation might be positively correlated with the cell cycle process leading to carcinogenesis, which is associated with poorer survival outcomes (**Supplementary Figure 2C**).

Next, we measured the intratumoral abundance of various immune cell populations with differential gene expression based on the nCounter immune profile panel. At the metagene level, the 14 immune cells composition were assessed, and the scores of some immune cell subsets, such as B cells (p=0.031), CD45^+^ cells (p=0.035), dendritic cells (p=0.024), macrophages (p=0.006), mast cells (p=0.009), neutrophils (p=0.031) and T cells (p=0.018), were significantly decreased in relapsed samples with *NOTCH1*-mutant compared to *NOTCH1* wild-type (**Figures 5C, D**), indicating a weakened immune phenotype in *NOTCH1*-mutant relapsed patients.

### Additional immune signatures in the *NOTCH1* mutation group

**Figure 6:**
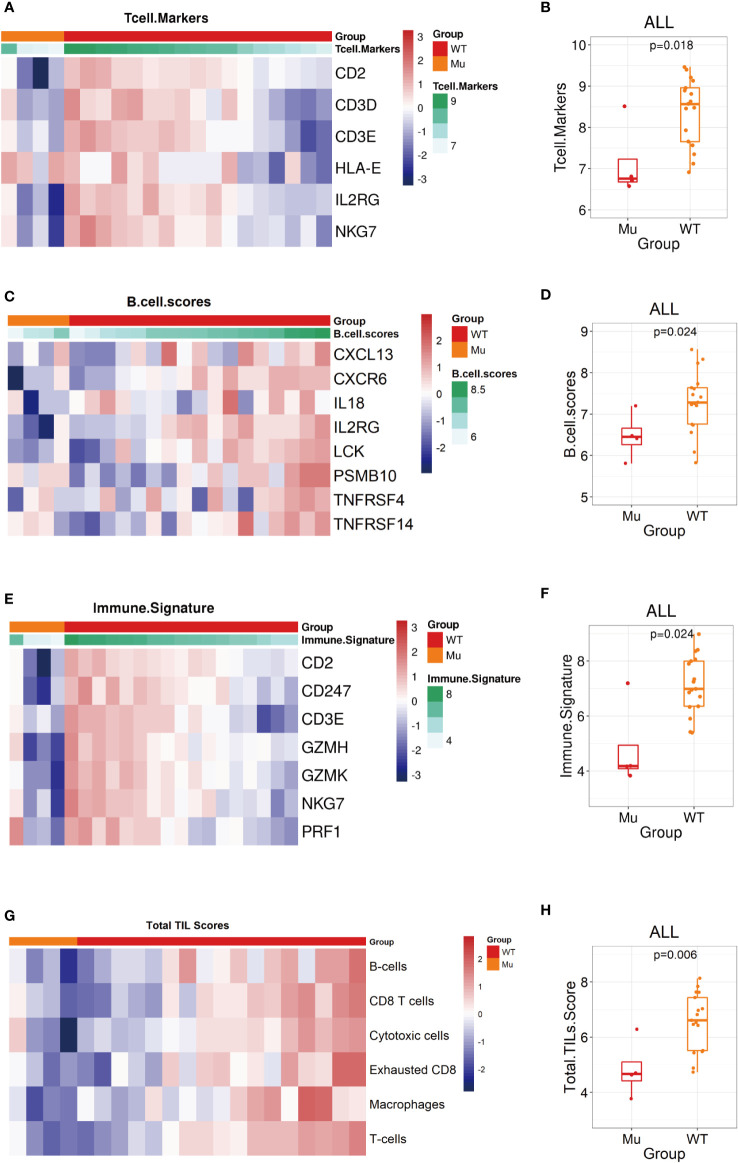
Biological signatures in the *NOTCH1* mutation group. **(A, B)** The T cell markers score assessment shown as heatmap and the statistic analysis in two groups. **(C, D)** The B cell markers score assessment shown as heatmap and the statistic analysis in two groups. **(D, E)** The immune signature score assessment shown as heatmap and the statistic analysis in two groups. **(G, H)** The total TILs score assessment shown as heatmap and the statistic analysis in two groups.

Several biomarkers have been developed to evaluate different aspects of the adaptive immune response, such as the downstream consequences of immune activation, as measured by the presence of immune cells, and the immunoscore or gene expression signature related to the immune environment (32). We next evaluated 10 predefined immune gene signatures included in the immuno-oncology panel to assess the biological differences in the tumor immune environment. The results showed that the expression of related gene signatures, such as the T-cells marker score (*CD2*, *CD3D*, *CD3E*, *HLA-E*, *IL2RG*, *NKG7*) (WT vs. Mu: 8.6 vs. 6.7, p=0.018, **Figures 6A, B**), B-cells score (*CXCL13*, *CXCR6*, *IL18*, *IL2RG*, *LCK*, *PSMB10*, *TNFRSF4*, *TNFRSF14*) (WT vs. Mu: 7.3 vs. 6.4, p=0.024, **Figures 6C, D**), and immune signature score (*CD2*, *CD247*, *CD3E*, *GZMH*, *GZMK*, *NKG7*, *PRF1*) (WT vs. Mu: 7.0 vs. 4.1, p=0.024, **Figures 6E, F**), were significantly reduced in recurrent patients with *NOTCH1* mutation. In addition, the TILs score, which incorporates different immune cell populations (B cells, CD8 T cells, cytotoxic cells, exhausted CD8 cells, macrophages, NK CD56dim cells, total T cells), was further analyzed and found to be significantly decreased in the *NOTCH1*-mutant group (WT vs. Mu: 6.6 vs. 4.5, p=0.006, **Figures 6G, H**). However, no significant differences were found in terms of the cytotoxic T lymphocyte activity (CTL) score (WT vs. Mu: 7.5 vs. 6.43, p=0.2), cytolytic activity (CYT) score (WT vs. Mu: 7.9 vs. 6.8, p=0.2), chemokine score (WT vs. Mu: 7.8 vs. 6.8, p=0.14), angiogenesis score (WT vs. Mu: 11.6 vs. 11.9, p=0.57), IFN-γ signature score (WT vs. Mu: 2.6 vs. 2.4, p=0.76), or T-cell-inflammatory gene expression profile (GEP) score (WT vs. Mu: -0.4 vs. -0.5, p=0.46) (**Supplementary Figures 2D–I**). These results highlighted that the attenuation of these immunophenotypes may lead to immunosurveillance escape and postoperative recurrence in patients with *NOTCH1* mutation.

### *NOTCH1* mutation status was correlated with immune infiltration in the laryngeal cancer datasets from TCGA

**Figure 7:**
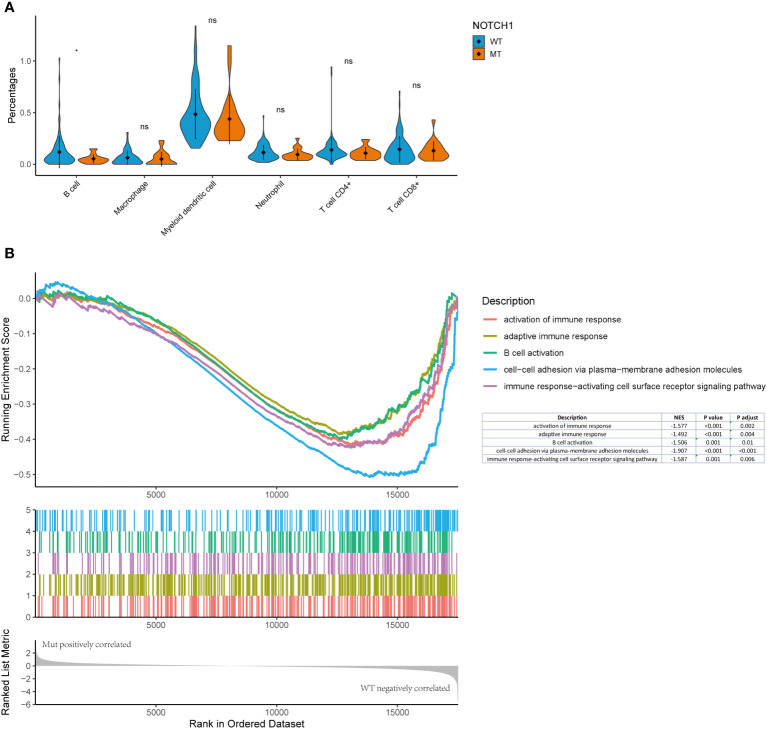
*NOTCH1* mutation status was correlated with immune infiltration in the laryngeal cancer datasets from TCGA. **(A)** TIMER algorithm to estimate the TCGA immune cell infiltration status in laryngeal cancer datasets. **(B)** The GSEA results revealed prominent enrichment in discrepant signatures.

To further confirm the alterations observed in the various immune cell populations, we used the TIMER algorithm to estimate the TCGA immune cell infiltration status in laryngeal cancer datasets. In line with the findings of the NanoString gene expression assay, the proportion of activated B cells in *NOTCH1*-mutant tumors was lower than that in wild-type tumors (**Figures 7A**). To further explore the potential mechanism behind *NOTCH1* mutation and the adaptive immune response in laryngeal cancer, immune-related signatures and signaling genes were analyzed based on RNA data from the TCGA database. The GSEA results revealed prominent enrichment in signatures related to the activation of the immune response pathway (NES: -1.57, adjusted p<0.001), adaptive immune response pathway (NES: -1.49, adjusted p<0.001), B-cell activation pathway (NES: -1.570, adjusted p=0.009) and immune response-activating cell surface receptor signaling pathway (NES: -1.58, adjusted p<0.001), all of which were downregulated in the *NOTCH1*-mutant group (**Figure 7B**), further indicating a weakened adaptive immune response. Moreover, cell-cell adhesion *via* the plasma membrane adhesion pathway (NES: -1.91, adjusted p<0.001) was also downregulated in the *NOTCH1*-mutant group (**Figure 7B**), highlighting the impaired cell-cell adhesion functions in these patients. These results suggest that preexisting tumor immunity characteristics are a determinant of recurrence, and the weakened adaptive immune response and impaired adhesion functions in the tumor microenvironment may contribute to recurrence.

## Discussion

Although the recurrence of early laryngeal cancer is relatively common after resection, the genetic and immunologic features of recurrence have not been well described. Our study is the first to identify the distinct molecular bases of laryngeal cancer recurrence and the immunologic characteristics of patients who experience recurrence events. The results showed that *NOTCH1* mutation was associated with decreased RFS due to a weakened immune response. This finding is consistent with the growing body of research showing that host immune response play a key role in tumorigenesis and cancer progression (30, 31).

In this study, we performed targeted DNA-NGS on tumor samples from 42 Chinese patients with early laryngeal cancer to analyzed their genomic profiles. We are the first to report on genomic mutations in early laryngeal cancers, which may be valuable for developing more precise treatments in the future. Based on previous studies of HNSCC and advanced laryngeal cancer (33, 34), we found similarities but also differences in the types of genomic changes. The mutation rate of *TP53* was the highest in all groups, the mutation rate in our cohort was much higher than that reported (63.1%) for HNSCC. However, for advanced laryngeal cancers, different studies reported diverse results on *TP53* mutation rate, like 90.0% and 38.1%. The *TP53* mutation rate in our results was close to 90.0%, this mutation rate (90%) was evaluated in 60 laryngeal cancer samples study. The latter one (38.1% mutation rate) may be due to the small sample size (only 21 samples) or technical reasons like insufficient sequencing depth or limited coverage of gene hot spots, etc. (35). The mutation frequency of *KMT2D* in advanced stages (33.3%) was significantly higher than that in early stages (7.0%), which may be due to the evolution of the genetics of tumor under selective pressures, suggesting that the stated role of the differences need be clarified in the treatment strategy depending on the disease stages. Compared with previous studies focusing on individual gene mutations and the effect on survival outcomes, NGS offers the possibility of high-throughput sequencing that can simultaneously detect a variety of novel genetic mutations (36). This study is the first to explore the association of NGS results with survival prognosis in patients with resected laryngeal cancer, which is one of the highlights of this study.

TMB has been shown to be a predictor of immunotherapy response (8, 9). To the best to our knowledge, we report for the first time that early laryngeal cancer patients with a higher TMB have a significantly higher recurrence rate, emphasizing that TMB-H is a poor prognostic factor for laryngeal cancer. In addition, patients with multiple somatic gene mutations (>1 mutation) had a worse RFS than patients with only one somatic gene mutation. These results suggest that TMB-H may be related to the degree of malignancy of laryngeal cancers, leading early recurrence in laryngeal cancer. The administration postoperative adjuvant therapy with immune checkpoint inhibitors to laryngeal cancer patients with TMB-H may contribute to a reduction in recurrence events and an improvement in their OS. In addition to TMB, *NOTCH1* mutations are also known to be oncogenic, and in a study focusing on oral squamous cell carcinoma and HNSCC cell lines, patients with tumors carrying *NOTCH1* mutation had significantly poorer survival outcomes (37). Inamura et al. also confirmed that *NOTCH1* regulates the invasion and metastasis of HNSCC by c-MYC-induced EMT (38). Inactivation of *NOTCH1*, as a tumor suppressor gene, may inhibit the Wnt/β-catenin signaling pathway, which is associated with cell polarity and differentiation (39, 40). Additionally, cell cycle-related genes were found to be enriched in the *NOTCH1*-mutatnt group, indicating that *NOTCH1* mutation was positively correlated with the cell cycle in the GSEA results. These results strongly suggest that *NOTCH1* mutation is associated with carcinogenesis and poorer survival outcomes, and potential implications of these mutations as biomarkers and interventional targets.

Several indicators have been identified that can characterize the potential of tumor cells to initiate an adaptive immune response, such as TMB and MSI (41), or the downstream consequences of immune activation, such as the presence of immune cells, the immune score and gene expression characteristics associated with the immune environment (32). A number of candidate gene expression signatures have been developed and evaluated in baseline tumor biopsy specimens from patients with metastatic melanoma and gastric cancer (42–45). The results of transcriptome analysis also revealed a series of changes in genomic and immunological characteristics that are associated with *NOTCH1* mutations. As expected, we found that several upregulated or downregulated genes in patients with recurrent laryngeal cancer with *NOTCH1* mutations have the potential to be small-molecule targets for precision therapy. In addition, several key immune response pathways are downregulated in recurrent tumors, which are typically characterized by weakened immunophenotypes, such as significantly reduced T and B-cell expression levels, leading to the decrease in TILs infiltration. In fact, the role of the *NOTCH* signaling pathway in T and B-cells has been described, and *NOTCH1* is an important mediator for NOTCH ligands to enhance B-cell activation and antibody secretion (46, 47). In our study, the GSEA results obtained from an analysis the TCGA-laryngeal cancer dataset also showed that gene sets associated with B-cell active pathways were significantly downregulated in tumors with *NOTCH1* mutations. Therefore, preexisting tumor immune characteristics may be a determinant of recurrence. This finding is consistent with the growing body of studies suggesting that the host immune response plays a key role in tumorigenesis and progression. As in studies on resected NSCLC, an impaired adaptive immune response is thought to be responsible for recurrent tumors (30, 31).

TILs have been proposed as crucial prognostic indicators in several cancer types (16–18), and the TIL density has greater predictive power for survival than the well-established TNM classification system in gastric cancer (19). Moreover, TILs as a biomarker were investigated gradually in HNSCC. Mann et al. reported an immune profile driven by CD103^+^ TIL content was associated with significantly improved survival outcomes and was a stronger predictor of survival in recurrent/persistent laryngeal squamous cell carcinomas (48). CD103^+^ TIL are mainly consisted of dendritic cells, which are unique hematopoietic cells, linking innate and adaptive immune response. Similar finding that recurrent tumors with *NOTCH1* mutation displayed decreased TILs score, along with reduced dendritic cell infiltration in our study. Both our two findings demonstrated the prognostic value of TILs in laryngeal cancers. In addition, Zhou et al. reported a combined analysis of the density of tumor-associated immune cells and their location may help predict patient survival in laryngeal cancers, like a high density of intratumoral CD68^+^ cells and peritumoral CD163^+^ cells were significantly associated with poor OS durations (49). Double CD68^+^ CD163^+^ cells mainly defined as M2 macrophages, and M2 macrophages are mainly involved in anti-inflammatory responses. These results fully highlighted that the attenuation of immunophenotypes may lead to immunosurveillance escape and postoperative recurrence in patients with *NOTCH1* mutation in our study. Thus, predictive models may prove valuable in prognostic stratification and lead to personalized treatment paradigms for this patient population, this finding is consistent with the fact that an effective host immune response plays a key role in tumorigenesis and cancer progression.

There are some limitations to this study, which should be noted. First, only 42 patients with stage T1-2N0 disease were analyzed in this study. In particular, only 21 patients with available sequencing data were included in the analysis of immunological characteristics. Larger cohorts of patients may lead to stronger biological conclusions. Second, the NanoString nCount technology used for immunosignature analysis is based on bulk RNA analysis, which provides a view of gene expression across the entire sample (50, 51). This method cannot investigate the alterations and characteristics of different immune cell populations at the *in situ* level.

In conclusion, we describe the unique tumor molecular bases and immune characteristics of recurrent tumors in patients with resected laryngeal carcinoma, which may help further elucidate the mechanisms of recurrence and thus aid in the development of potential therapeutic strategies for these patients. Obviously, these findings must be prospectively validated in the future.

## Data availability statement

The sequencing datasets presented in this article are not readily available because human genetic resource data should not be publicly available due to the Regulations on Management of Human Genetic Resource (Guo Ling No.71) in China. The datasets used during the current study are available from the corresponding author on reasonable request, requests to access the datasets should be directed to X. Chen, chenxi_njmu@126.com.

## Ethics statement

The studies involving human participants were reviewed and approved by The First Affiliated Hospital, Nanjing Medical University. The patients/participants provided their written informed consent to participate in this study.

## Author contributions

There are 4 first authors in this manuscript and they have equally contributed to this project. XG, HC, LZ and DC were responsible for the design of the project and all data sorting, and writing articles. WL, and DC were responsible for data analysis and writing articles. JX, HZ, and LZ were responsible for imaging evaluation. YS, LZ,WD,QC were responsible for sample sequencing and data analysis. XW, XC supervised the work. All authors read and approved the final manuscript.

## Funding

This work was supported by grants from the National Natural Science Foundation of China (grant numbers 81972763).

## Conflict of interest

Author Dongsheng Chen, Le-le Zhao, Yun-jie Song, Ming-zhe Xiao, Wang-long Deng and Chuang Qi are employed by The State Key Laboratory of Translational Medicine and Innovative Drug Development, Medical Department, Jiangsu Simcere Diagnostics Co., Ltd., Nanjing, China.

The remaining authors declare that the research was conducted in the absence of any commercial or financial relationships that could be constructed as a potential conflict of interest.

## Publisher’s note

All claims expressed in this article are solely those of the authors and do not necessarily represent those of their affiliated organizations, or those of the publisher, the editors and the reviewers. Any product that may be evaluated in this article, or claim that may be made by its manufacturer, is not guaranteed or endorsed by the publisher.

## Supplementary material

Supplementary Figure 1 Mutation analysis of laryngeal cancer patients. **(A)** Co-barblot analysis in the cohort. **(B)** K-M survival curve on number of mutations with PFS.

Supplementary Figure 2 Pathways and additional biological signatures in the *NOTCH1* mutation group. **(A, B)** GO and KEGG analysis on DEGs **(C)** the function of DEGs by GSEA analysis. **(D–I)** The CTL score, CYT score, chemokines score, angiogenesis score, IFN-γ score and GEP score assessment shown as heatmap and the statistic analysis in two groups.
