# Supplementary figures and images for: NOTCH1 mutation associates with impaired immune response and decreased relapse-free survival in patients with resected T1-2N0 laryngeal cancer
Source: Front Immunol. 2022 Jul 15;13:920253. doi: 10.3389/fimmu.2022.920253 (PMC9336464; doi:10.3389/fimmu.2022.920253)

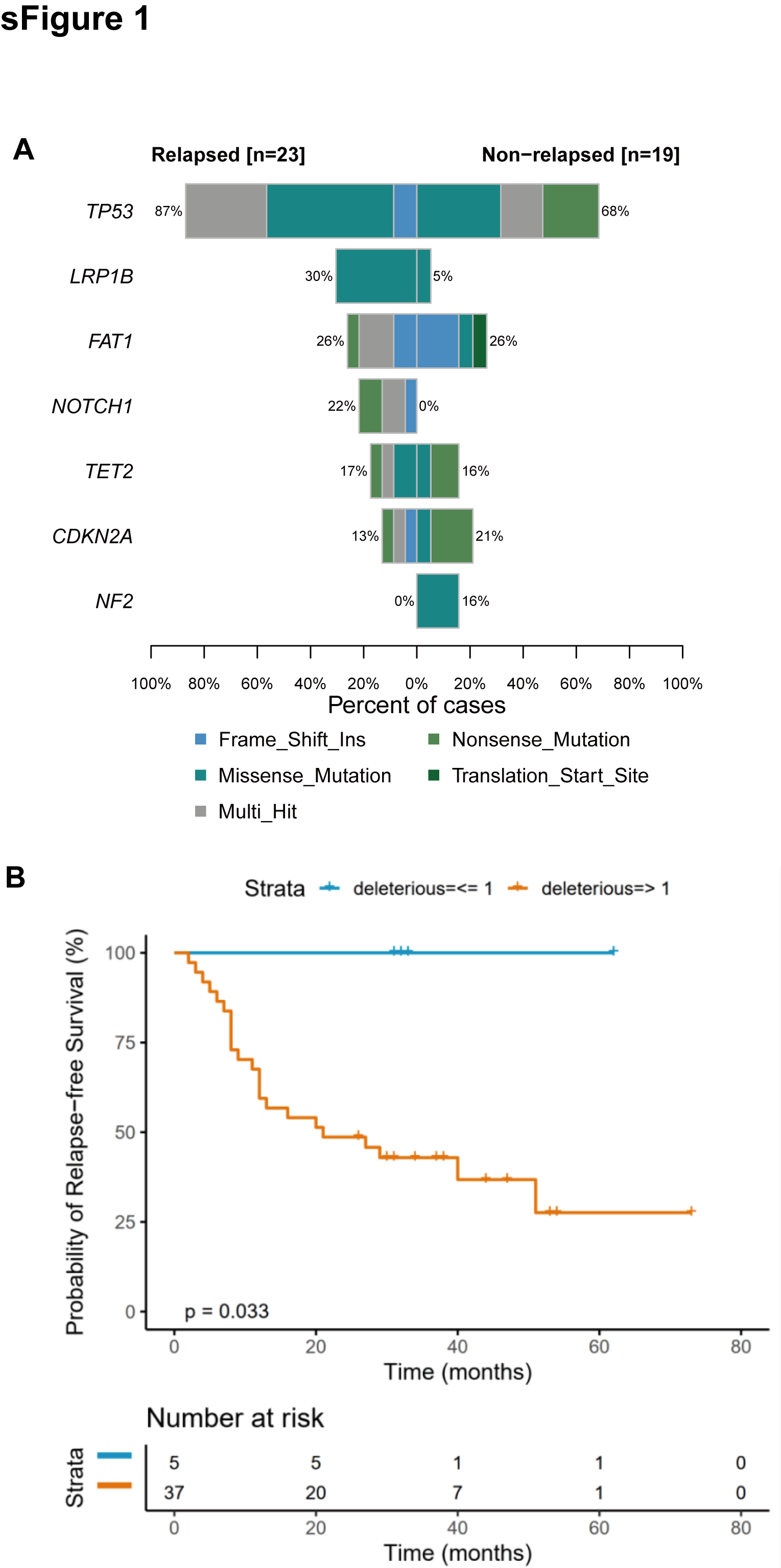

Supplement: Supplementary Figure 1 — Mutation analysis of laryngeal cancer patients. (A) Co-barblot analysis in the cohort. (B) K-M survival curve on number of mutations with PFS. [file Image_1.tif]

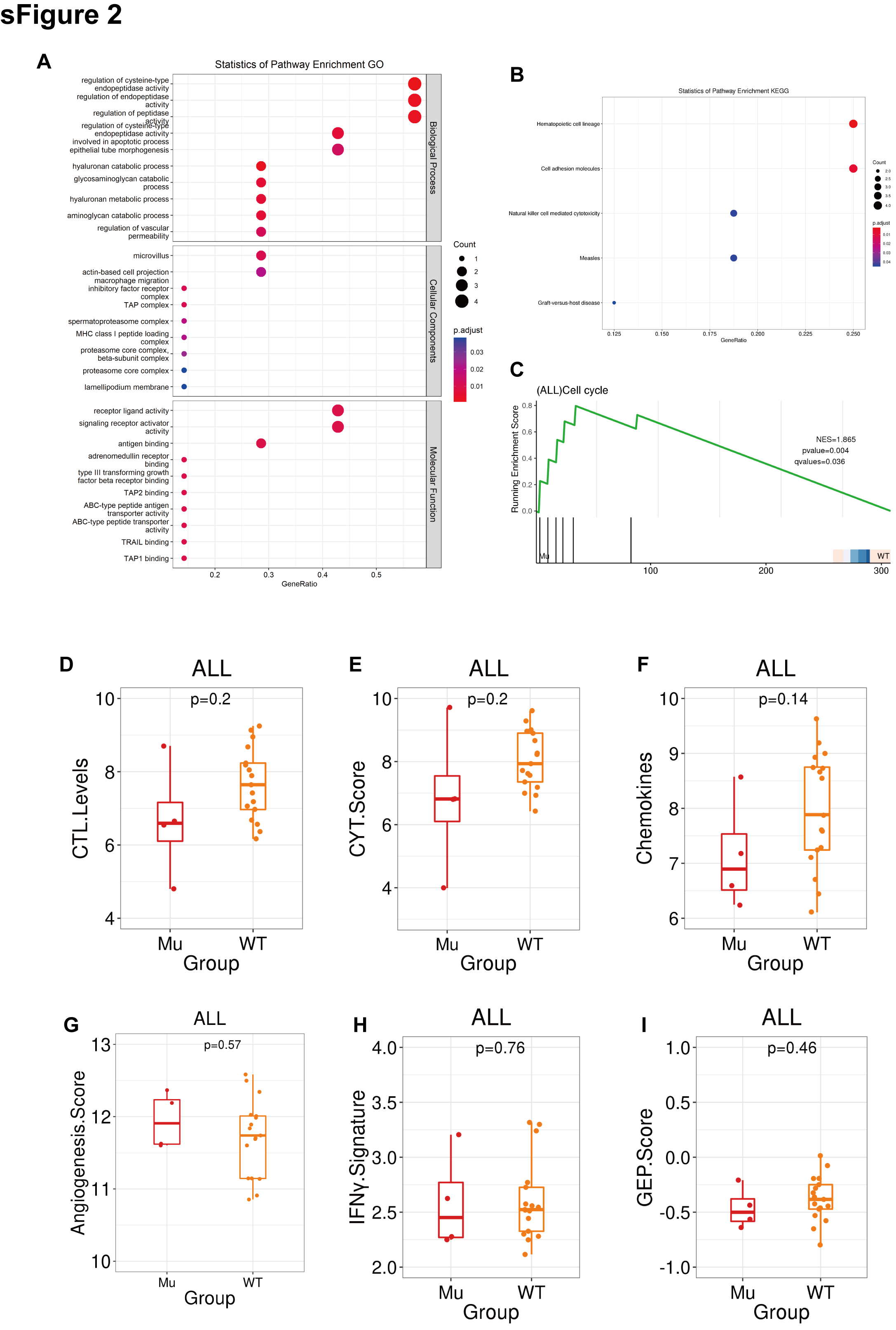

Supplement: Supplementary Figure 2 — Pathways and additional biological signatures in the NOTCH1 mutation group. (A, B) GO and KEGG analysis on DEGs (C) the function of DEGs by GSEA analysis. (D–I) The CTL score, CYT score, chemokines score, angiogenesis score, IFN-γ score and GEP score assessment shown as heatmap and the statistic analysis in two groups. [file Image_2.tif]
