# Supplementary material for: NOTCH1 mutation associates with impaired immune response and decreased relapse-free survival in patients with resected T1-2N0 laryngeal cancer
Source: Front Immunol. 2022 Jul 15;13:920253. doi: 10.3389/fimmu.2022.920253 (PMC9336464; doi:10.3389/fimmu.2022.920253)
Supplement: Supplementary file 3 [file Table_1.docx]

**sTable 1: TME 289 gene list**

| **ABCF1** | **CD44** | **FCGR1A** | **IL21R** | **NFKBIA** | **TIE1** |
| --- | --- | --- | --- | --- | --- |
| **ADM** | **CD47** | **FCGR2B** | **IL2RA** | **NKG7** | **TIGIT** |
| **ADORA2A** | **CD48** | **FCRL2** | **IL2RB** | **NOS2** | **TLR3** |
| **AKT1** | **CD6** | **FGF13** | **IL2RG** | **NT5E** | **TLR7** |
| **ANGPT2** | **CD68** | **FOXP3** | **IL4** | **OAS1** | **TLR8** |
| **ARG1** | **CD69** | **FPR1** | **IL6** | **OAS2** | **TLR9** |
| **ATM** | **CD70** | **FUT4** | **IL7R** | **OAS3** | **TNF** |
| **AXL** | **CD74** | **G6PD** | **IRF1** | **PDCD1** | **TNFRSF14** |
| **BCL2** | **CD79A** | **GBP1** | **IRF4** | **PDCD1LG2** | **TNFRSF17** |
| **BIRC5** | **CD79B** | **GNLY** | **IRF9** | **PDGFA** | **TNFRSF18** |
| **BLK** | **CD80** | **GUSB** | **ISG15** | **PDGFB** | **TNFRSF1A** |
| **BLM** | **CD84** | **GZMA** | **ITGA1** | **PECAM1** | **TNFRSF1B** |
| **BRCA1** | **CD86** | **GZMB** | **ITGAE** | **PIK3CA** | **TNFRSF4** |
| **BRCA2** | **CD8A** | **GZMH** | **ITGAL** | **PIK3CD** | **TNFRSF9** |
| **BRIP1** | **CD8B** | **GZMK** | **ITGAM** | **PMS2** | **TNFSF10** |
| **BTLA** | **CDKN2A** | **HAVCR2** | **ITGAX** | **PNOC** | **TNFSF13B** |
| **C1QA** | **CEACAM3** | **HDC** | **ITGB2** | **POLR2A** | **TNFSF18** |
| **C1QB** | **CMKLR1** | **HERC6** | **KIR2DL3** | **PRF1** | **TNFSF4** |
| **CCL13** | **CPA3** | **HIF1A** | **KIR3DL1** | **PSMB10** | **TNFSF9** |
| **CCL18** | **CSF1R** | **HLA-DMA** | **KIR3DL2** | **PSMB9** | **TRAT1** |
| **CCL2** | **CSF2** | **HLA-DMB** | **KLRB1** | **PTEN** | **TWIST1** |
| **CCL20** | **CSF2RB** | **HLA-DOA** | **KLRD1** | **PTGER4** | **VCAM1** |
| **CCL21** | **CSF3R** | **HLA-DOB** | **KLRK1** | **PTGS2** | **VEGFA** |
| **CCL22** | **CTAG1B** | **HLA-DPA1** | **LAG3** | **PTPN11** | **VTCN1** |
| **CCL4** | **CTLA4** | **HLA-DQA2** | **LCK** | **PTPRC** | **ZAP70** |
| **CCL5** | **CTSS** | **HLA-DRA** | **LILRB2** | **PVR** | **ZEB1** |
| **CCL7** | **CTSW** | **HSD11B1** | **LY9** | **RAD51** | **CXCL2** |
| **CCND1** | **CX3CL1** | **ICAM1** | **LYZ** | **RB1** | **FCGR3B** |
| **CCR2** | **CX3CR1** | **ICOS** | **MAGEA1** | **RORC** | **GZMM** |
| **CCR4** | **CXCL1** | **ICOSLG** | **MAGEA12** | **RUNX3** | **HLA-DQA1** |
| **CCR5** | **CXCL10** | **IDO1** | **MAGEA4** | **S100A12** | **HLA-DRB1** |
| **CD14** | **CXCL11** | **IFI27** | **MAGEC2** | **S100A8** | **HLA-E** |
| **CD163** | **CXCL12** | **IFI35** | **MELK** | **S100A9** | **OAZ1** |
| **CD19** | **CXCL13** | **IFI6** | **MKI67** | **SDHA** | **PF4** |
| **CD1C** | **CXCL5** | **IFIH1** | **MLANA** | **SELL** | **PRR5** |
| **CD2** | **CXCL8** | **IFIT1** | **MLH1** | **SH2D1A** | **STK11IP** |
| **CD209** | **CXCL9** | **IFIT2** | **MMP9** | **SIGLEC5** | **TBC1D10B** |
| **CD244** | **CXCR2** | **IFIT3** | **MRC1** | **SLAMF7** | **TPSAB1** |
| **CD247** | **CXCR3** | **IFITM1** | **MS4A1** | **SNAI1** | **UBB** |
| **CD27** | **CXCR4** | **IFITM2** | **MS4A2** | **SPIB** |  |
| **CD274** | **CXCR6** | **IFNG** | **MS4A4A** | **STAT1** |  |
| **CD276** | **CYBB** | **IL10** | **MSH2** | **STAT3** |  |
| **CD28** | **DLL4** | **IL10RA** | **MSH6** | **STAT4** |  |
| **CD38** | **EGFR** | **IL12RB2** | **MTOR** | **TAP1** |  |
| **CD3D** | **EIF2AK2** | **IL15** | **MX1** | **TBP** |  |
| **CD3E** | **ENTPD1** | **IL17A** | **MYC** | **TBX21** |  |
| **CD3G** | **EOMES** | **IL18** | **NBN** | **TCL1A** |  |
| **CD4** | **FAS** | **IL1A** | **NCAM1** | **TDO2** |  |
| **CD40** | **FASLG** | **IL1B** | **NCR1** | **TFRC** |  |
| **CD40LG** | **FCAR** | **IL2** | **NECTIN2** | **TGFB1** |  |
