# Supplementary material for: NOTCH1 mutation associates with impaired immune response and decreased relapse-free survival in patients with resected T1-2N0 laryngeal cancer
Source: Front Immunol. 2022 Jul 15;13:920253. doi: 10.3389/fimmu.2022.920253 (PMC9336464; doi:10.3389/fimmu.2022.920253)
Supplement: Supplementary file 4 [file Table_2.docx]

**sTable 2: Cell type gene list**

| **Cell type** | **Gene list** |
| --- | --- |
| T cells | CD3D、CD3E、CD3G、CD6、SH2D1A、TRAT1 |
| B cells | BLK、CD19、FCRL2、MS4A1、PNOC、SPIB、TCL1A、TNFRSF17 |
| Mast cells | CPA3、HDC、MS4A2 |
| DC | CCL13、CD209、HSD11B1 |
| Macrophages | CD163、CD68、CD84、MS4A4A |
| Neutrophils | CEACAM3、CSF3R、FCAR、FPR1、S100A12、SIGLEC5 |
| Cytotoxic cells | CTSW、GNLY、GZMA/B/H、KLRB1、KLRD1、KLRK1、NKG7、PRF1 |
| Exhausted CD8 | PTGER4、LAG3、EOMES、CD244 |
| NK CD56 cell | KIR3DL1/2/3、IL21R |
| CD8 T cell | CD8B、CD8A |
| CD45 cell | PTPRC |
| Th1 cell | TBX21 |
| NK cell | NCR1 |
| Treg cell | FOXP3 |
