# Supplementary material for: NOTCH1 mutation associates with impaired immune response and decreased relapse-free survival in patients with resected T1-2N0 laryngeal cancer
Source: Front Immunol. 2022 Jul 15;13:920253. doi: 10.3389/fimmu.2022.920253 (PMC9336464; doi:10.3389/fimmu.2022.920253)
Supplement: Supplementary file 5 [file Table_3.docx]

**sTable 3: Signatures/Scores gene list and respective references**

| **Signatures/Scores** | **Gene/cell list** | **References** |
| --- | --- | --- |
| T cell score | CD2, CD3D, CD3E, HLA-E, IL2RG, NKG7 | PMID: 28650338 |
| B cell score | CXCL13, CXCR6, IL18, IL2RG, LCK, PSMB10, TNFRSF4, TNFRSF14 | PMID: 34921229 |
| Immune signature | CD2, CD247, CD3E, GZMH, GZMK, NKG7, PRF1 | PMID: 28104840 |
| CTL score | CD8A, CD8B, GZMA, GZMB, PRF1 | PMID: 30127393 |
| CYT score | PRF1, GZMA | PMID: 25594174 |
| Chemokines score | CCL5, CCR5, CXCL9, CXCR6 | PMID: 18178862 |
| Angiogenesis score | VEGFA, PECAM1 | PMID: 21778317 |
| IFN-γ signature | CXCL10, CXCL9, HLA-DRA, IDO1, IFNG, STAT1 | PMID: 28650338 |
| T cell inflamed GEP score | CCL5, CD27, CD274, CD276, CD8A, CMKLR1, CXCL9, CXCR6, HLA-DQA1, HLA-DRB1, HLA-E, IDO1, LAG3, NKG7, PDCD1, PSMB10, STAT1, TIGIT | PMID: 28650338 |
| Total TILs score | B-cells, CD8 T cells, Cytotoxic cells, Exhausted CD8, Macrophages, NK CD56dim cells, T-cells | PMID: 28239471 |
