# Supplementary material for: NOTCH1 mutation associates with impaired immune response and decreased relapse-free survival in patients with resected T1-2N0 laryngeal cancer
Source: Front Immunol. 2022 Jul 15;13:920253. doi: 10.3389/fimmu.2022.920253 (PMC9336464; doi:10.3389/fimmu.2022.920253)
Supplement: Supplementary file 6 [file Table_4.docx]

**sTable 4: TSO500 gene list**

| **ABL1** | **CCND3** | **EPCAM** | **FOXA1** | **IGF1** | **MEN1** | **PDGFRB** | **REL** | **TAF1** |
| --- | --- | --- | --- | --- | --- | --- | --- | --- |
| **ABL2** | **CCNE1** | **EPHA3** | **FOXL2** | **IGF1R** | **MET** | **PDK1** | **RET** | **TBX3** |
| **ACVR1** | **CD274** | **EPHA5** | **FOXO1** | **IGF2** | **MGA** | **PDPK1** | **RFWD2** | **TCEB1** |
| **ACVR1B** | **CD276** | **EPHA7** | **FOXP1** | **IKBKE** | **MITF** | **PGR** | **RHEB** | **TCF3** |
| **AKT1** | **CD74** | **EPHB1** | **FRS2** | **IKZF1** | **MLH1** | **PHF6** | **RHOA** | **TCF7L2** |
| **AKT2** | **CD79A** | **ERBB2** | **FUBP1** | **IL10** | **MLL** | **PHOX2B** | **RICTOR** | **TERC** |
| **AKT3** | **CD79B** | **ERBB3** | **FYN** | **IL7R** | **MLLT3** | **PIK3C2B** | **RIT1** | **TERT** |
| **ALK** | **CDC73** | **ERBB4** | **GABRA6** | **INHA** | **MPL** | **PIK3C2G** | **RNF43** | **TET1** |
| **ALOX12B** | **CDH1** | **ERCC1** | **GATA1** | **INHBA** | **MRE11A** | **PIK3C3** | **ROS1** | **TET2** |
| **ANKRD11** | **CDK12** | **ERCC2** | **GATA2** | **INPP4A** | **MSH2** | **PIK3CA** | **RPS6KA4** | **TFE3** |
| **ANKRD26** | **CDK4** | **ERCC3** | **GATA3** | **INPP4B** | **MSH3** | **PIK3CB** | **RPS6KB1** | **TFRC** |
| **APC** | **CDK6** | **ERCC4** | **GATA4** | **INSR** | **MSH6** | **PIK3CD** | **RPS6KB2** | **TGFBR1** |
| **AR** | **CDK8** | **ERCC5** | **GATA6** | **IRF2** | **MST1** | **PIK3CG** | **RPTOR** | **TGFBR2** |
| **ARAF** | **CDKN1A** | **ERG** | **GEN1** | **IRF4** | **MST1R** | **PIK3R1** | **RUNX1** | **TMEM127** |
| **ARFRP1** | **CDKN1B** | **ERRFI1** | **GID4** | **IRS1** | **MTOR** | **PIK3R2** | **RUNX1T1** | **TMPRSS2** |
| **ARID1A** | **CDKN2A** | **ESR1** | **GLI1** | **IRS2** | **MUTYH** | **PIK3R3** | **RYBP** | **TNFAIP3** |
| **ARID1B** | **CDKN2B** | **ETS1** | **GNA11** | **JAK1** | **MYB** | **PIM1** | **SDHA** | **TNFRSF14** |
| **ARID2** | **CDKN2C** | **ETV1** | **GNA13** | **JAK2** | **MYC** | **PLCG2** | **SDHAF2** | **TOP1** |
| **ARID5B** | **CEBPA** | **ETV4** | **GNAQ** | **JAK3** | **MYCL1** | **PLK2** | **SDHB** | **TOP2A** |
| **ASXL1** | **CENPA** | **ETV5** | **GNAS** | **JUN** | **MYCN** | **PMAIP1** | **SDHC** | **TP53** |
| **ASXL2** | **CHD2** | **ETV6** | **GPR124** | **KAT6A** | **MYD88** | **PMS1** | **SDHD** | **TP63** |
| **ATM** | **CHD4** | **EWSR1** | **GPS2** | **KDM5A** | **MYOD1** | **PMS2** | **SETBP1** | **TRAF2** |
| **ATR** | **CHEK1** | **EZH2** | **GREM1** | **KDM5C** | **NAB2** | **PNRC1** | **SETD2** | **TRAF7** |
| **ATRX** | **CHEK2** | **FAM123B** | **GRIN2A** | **KDM6A** | **NBN** | **POLD1** | **SF3B1** | **TSC1** |
| **AURKA** | **CIC** | **FAM175A** | **GRM3** | **KDR** | **NCOA3** | **POLE** | **SH2B3** | **TSC2** |
| **AURKB** | **CREBBP** | **FAM46C** | **GSK3B** | **KEAP1** | **NCOR1** | **PPARG** | **SH2D1A** | **TSHR** |
| **AXIN1** | **CRKL** | **FANCA** | **H3F3A** | **KEL** | **NEGR1** | **PPM1D** | **SHQ1** | **U2AF1** |
| **AXIN2** | **CRLF2** | **FANCC** | **H3F3B** | **KIF5B** | **NF1** | **PPP2R1A** | **SLIT2** | **VEGFA** |
| **AXL** | **CSF1R** | **FANCD2** | **H3F3C** | **KIT** | **NF2** | **PPP2R2A** | **SLX4** | **VHL** |
| **B2M** | **CSF3R** | **FANCE** | **HGF** | **KLF4** | **NFE2L2** | **PPP6C** | **SMAD2** | **VTCN1** |
| **BAP1** | **CSNK1A1** | **FANCF** | **HIST1H1C** | **KLHL6** | **NFKBIA** | **PRDM1** | **SMAD3** | **WISP3** |
| **BARD1** | **CTCF** | **FANCG** | **HIST1H2BD** | **KMT2B** | **NKX2-1** | **PREX2** | **SMAD4** | **WT1** |
| **BBC3** | **CTLA4** | **FANCI** | **HIST1H3A** | **KMT2C** | **NKX3-1** | **PRKAR1A** | **SMARCA4** | **XIAP** |
| **BCL10** | **CTNNA1** | **FANCL** | **HIST1H3B** | **KMT2D** | **NOTCH1** | **PRKCI** | **SMARCB1** | **XPO1** |
| **BCL2** | **CTNNB1** | **FAS** | **HIST1H3C** | **KRAS** | **NOTCH2** | **PRKDC** | **SMARCD1** | **XRCC2** |
| **BCL2L1** | **CUL3** | **FAT1** | **HIST1H3D** | **LAMP1** | **NOTCH3** | **PRSS8** | **SMC1A** | **YAP1** |
| **BCL2L11** | **CUX1** | **FBXW7** | **HIST1H3E** | **LATS1** | **NOTCH4** | **PTCH1** | **SMC3** | **YES1** |
| **BCL2L2** | **CXCR4** | **FGF1** | **HIST1H3F** | **LATS2** | **NPM1** | **PTEN** | **SMO** | **ZBTB2** |
| **BCL6** | **CYLD** | **FGF10** | **HIST1H3G** | **LMO1** | **NRAS** | **PTPN11** | **SNCAIP** | **ZBTB7A** |
| **BCOR** | **DAXX** | **FGF14** | **HIST1H3H** | **LRP1B** | **NRG1** | **PTPRD** | **SOCS1** | **ZFHX3** |
| **BCORL1** | **DCUN1D1** | **FGF19** | **HIST1H3I** | **LYN** | **NSD1** | **PTPRS** | **SOX10** | **ZNF217** |
| **BCR** | **DDR2** | **FGF2** | **HIST1H3J** | **LZTR1** | **NTRK1** | **PTPRT** | **SOX17** | **ZNF703** |
| **BIRC3** | **DDX41** | **FGF23** | **HIST2H3A** | **MAGI2** | **NTRK2** | **QKI** | **SOX2** | **ZRSR2** |
| **BLM** | **DHX15** | **FGF3** | **HIST2H3C** | **MALT1** | **NTRK3** | **RAB35** | **SOX9** |  |
| **BMPR1A** | **DICER1** | **FGF4** | **HIST2H3D** | **MAP2K1** | **NUP93** | **RAC1** | **SPEN** |  |
| **BRAF** | **DIS3** | **FGF5** | **HIST3H3** | **MAP2K2** | **NUTM1** | **RAD21** | **SPOP** |  |
| **BRCA1** | **DNAJB1** | **FGF6** | **HLA-A** | **MAP2K4** | **PAK1** | **RAD50** | **SPTA1** |  |
| **BRCA2** | **DNMT1** | **FGF7** | **HLA-B** | **MAP3K1** | **PAK3** | **RAD51** | **SRC** |  |
| **BRD4** | **DNMT3A** | **FGF8** | **HLA-C** | **MAP3K13** | **PAK7** | **RAD51B** | **SRSF2** |  |
| **BRIP1** | **DNMT3B** | **FGF9** | **HNF1A** | **MAP3K14** | **PALB2** | **RAD51C** | **STAG1** |  |
| **BTG1** | **DOT1L** | **FGFR1** | **HNRNPK** | **MAP3K4** | **PARK2** | **RAD51D** | **STAG2** |  |
| **BTK** | **E2F3** | **FGFR2** | **HOXB13** | **MAPK1** | **PARP1** | **RAD52** | **STAT3** |  |
| **C11orf30** | **EED** | **FGFR3** | **HRAS** | **MAPK3** | **PAX3** | **RAD54L** | **STAT4** |  |
| **CALR** | **EGFL7** | **FGFR4** | **HSD3B1** | **MAX** | **PAX5** | **RAF1** | **STAT5A** |  |
| **CARD11** | **EGFR** | **FH** | **HSP90AA1** | **MCL1** | **PAX7** | **RANBP2** | **STAT5B** |  |
| **CASP8** | **EIF1AX** | **FLCN** | **ICOSLG** | **MDC1** | **PAX8** | **RARA** | **STK11** |  |
| **CBFB** | **EIF4A2** | **FLI1** | **ID3** | **MDM2** | **PBRM1** | **RASA1** | **STK40** |  |
| **CBL** | **EIF4E** | **FLT1** | **IDH1** | **MDM4** | **PDCD1** | **RB1** | **SUFU** |  |
| **CCND1** | **EML4** | **FLT3** | **IDH2** | **MED12** | **PDCD1LG2** | **RBM10** | **SUZ12** |  |
| **CCND2** | **EP300** | **FLT4** | **IFNGR1** | **MEF2B** | **PDGFRA** | **RECQL4** | **SYK** |  |
