# Supplementary material for: NOTCH1 mutation associates with impaired immune response and decreased relapse-free survival in patients with resected T1-2N0 laryngeal cancer
Source: Front Immunol. 2022 Jul 15;13:920253. doi: 10.3389/fimmu.2022.920253 (PMC9336464; doi:10.3389/fimmu.2022.920253)
Supplement: Supplementary file 7 [file Table_5.docx]

**sTable 5：univariate and multivariate analysis on NOTCH1 mutation with OS**

|  | Univariable | | | | Multivariable | | |
| --- | --- | --- | --- | --- | --- | --- | --- |
| Characteristic |  | HR^1^ | 95% CI^1^ | p-value | HR^1^ | 95% CI^1^ | p-value |
| Sex |  |  |  |  |  |  |  |
| Female |  | — | — |  |  |  |  |
| Male |  | 0.72 | 0.09, 6.00 | 0.8 |  |  |  |
| Somking_history |  |  |  |  |  |  |  |
| Non_smoker |  | — | — |  | — | — |  |
| Light_smoker |  | 3.52 | 0.64, 19.3 | 0.15 | 4.42 | 0.76, 25.7 | 0.10 |
| Heavy_smoker |  | 0.94 | 0.09, 10.4 | >0.9 | 0.58 | 0.05, 6.81 | 0.7 |
| Drinking_history |  |  |  |  |  |  |  |
| Never |  | — | — |  |  |  |  |
| Former |  | 0.94 | 0.10, 9.05 | >0.9 |  |  |  |
| Always |  | 2.32 | 0.52, 10.4 | 0.3 |  |  |  |
| Stage |  |  |  |  |  |  |  |
| T1 |  | — | — |  |  |  |  |
| T2 |  | 1.74 | 0.35, 8.70 | 0.5 |  |  |  |
| Anterior_commissure_involvement |  |  |  |  |  |  |  |
| No |  | — | — |  |  |  |  |
| Yes |  | 0.43 | 0.10, 1.81 | 0.2 |  |  |  |
| LRP1B |  |  |  |  |  |  |  |
| WT |  | — | — |  |  |  |  |
| MU |  | 1.14 | 0.23, 5.65 | 0.9 |  |  |  |
| NOTCH1 |  |  |  |  |  |  |  |
| WT |  | — | — |  | — | — |  |
| MU |  | 5.37 | 0.97, 29.6 | 0.054 | 10.2 | 1.49, 69.7 | 0.018 |
| TMB_level |  |  |  |  |  |  |  |
| TMB-L |  | — | — |  |  |  |  |
| TMB-H |  | 1.92 | 0.47, 7.83 | 0.4 |  |  |  |
| Age_group |  |  |  |  |  |  |  |
| < 65 |  | — | — |  |  |  |  |
| >= 65 |  | 1,490,867,834 | 0.00, Inf | >0.9 |  |  |  |
| ^1^HR = Hazard Ratio, CI = Confidence Interval | | | | | | | |
